# Impact of inactivated COVID-19 vaccines on lung injury in B.1.617.2 (Delta) variant-infected patients

**DOI:** 10.1186/s12941-023-00569-z

**Published:** 2023-03-21

**Authors:** Miao Lai, Kai Wang, Chengyuan Ding, Yi Yin, Xiaoling lin, Chuanjun Xu, Zhiliang Hu, Zhihang Peng

**Affiliations:** 1 School of Public Health, Nanjing Medical University, 101 Longmian Ave, Nanjing, 211166 China; 2 The First Affiliated Hospital of Nanjing Medical University, Nanjing, 210029 Jiangsu China; 3 Center for Global Health, School of Public Health, Nanjing Medical University, Nanjing, 211166 China; 4 Department of Radiology, The Second Hospital of Nanjing, Nanjing University of Chinese Medicine, Nanjing, 210003 China; 5 Department of Infectious Diseases, The Second Hospital of Nanjing, Nanjing University of Chinese Medicine, Nanjing, 210003 China

**Keywords:** COVID-19, COVID-19 vaccines, Lung injury, Artificial intelligence (AI), Chest CT

## Abstract

**Background:**

Chest computerized tomography (CT) scan is an important strategy that quantifies the severity of COVID-19 pneumonia. To what extent inactivated COVID-19 vaccines could impact the COVID-19 pneumonia on chest CT is not clear.

**Methods:**

This study recruited 357 SARS-COV-2 B.1.617.2 (Delta) variant-infected patients admitted to the Second Hospital of Nanjing from July to August 2021. An artificial intelligence-assisted CT imaging system was used to quantify the severity of COVID-19 pneumonia. We compared the volume of infection (VOI), percentage of infection (POI) and chest CT scores among patients with different vaccination statuses.

**Results:**

Of the 357 Delta variant-infected patients included for analysis, 105 were unvaccinated, 72 were partially vaccinated and 180 were fully vaccinated. Fully vaccination had the least lung injuries when quantified by VOI (median VOI of 222.4 cm^3^, 126.6 cm^3^ and 39.9 cm^3^ in unvaccinated, partially vaccinated and fully vaccinated, respectively; p < 0.001), POI (median POI of 7.60%, 3.55% and 1.20% in unvaccinated, partially vaccinated and fully vaccinated, respectively; p < 0.001) and chest CT scores (median CT score of 8.00, 6.00 and 4.00 in unvaccinated, partially vaccinated and fully vaccinated, respectively; p < 0.001). After adjustment for age, sex, comorbidity, time from illness onset to hospitalization and viral load, fully vaccination but not partial vaccination was significantly associated with less lung injuries quantified by VOI {adjust coefficient[95%CI] for “full vaccination”: − 106.10(− 167.30,44.89); p < 0.001}, POI {adjust coefficient[95%CI] for “full vaccination”: − 3.88(− 5.96, − 1.79); p = 0.001} and chest CT scores {adjust coefficient[95%CI] for “full vaccination”: − 1.81(− 2.72, − 0.91); p < 0.001}. The extent of reduction of pulmonary injuries was more profound in fully vaccinated patients with older age, having underlying diseases, and being female sex, as demonstrated by relatively larger absolute values of adjusted coefficients. Finally, even within the non-severe COVID-19 population, fully vaccinated patients were found to have less lung injuries.

**Conclusion:**

Fully vaccination but not partially vaccination could significantly protect lung injury manifested on chest CT. Our study provides additional evidence to encourage a full course of vaccination.

**Supplementary Information:**

The online version contains supplementary material available at 10.1186/s12941-023-00569-z.

## Introduction

COVID-19 caused by SARS-CoV-2 virus has become one of the most important public health problems worldwide [1]. COVID-19 vaccines have been proved to be effective against severe outcomes [2–6]. The categorized clinical outcomes (for examples, dichotomous outcomes of severe and non-severe diseases) in previous studies often do not encapsulate the complexity of the clinical status, and substantial heterogeneity of disease severity exists within each category of the clinical outcomes. When all patients fall into the same category, it is difficult to evaluate the effectiveness of COVID-19 vaccines since differences would not be expected between vaccinated and unvaccinated patients. A quantitative clinical outcome could maximally capture the variability of patients’ clinical status and this outcome parameter could also enable quantification of the protection exerted by vaccines. Studies have attempted to quantify the severity of COVID-19 through evaluation of the pneumonia on chest computerized tomography (CT) [7]. After assigning each lung lobe with a score based on the degree of pneumonia, the total chest CT score is calculated as the sum of 5 lobe scores [1, 8–11]. Studies have showed that semi-quantitative chest CT scores were associated with categorized COVID-19 outcomes, and higher chest CT score was predictive of adverse outcomes [12–15].

In the present study, using COVID-19 pneumonia on chest CT scan as a quantitative clinical outcome, we investigated the impact of inactivated COVID-19 vaccines on lung injury in B.1.617.2 (Delta) variant-infected patients. Since visual assessment of lesion extent even by experienced radiologists may be a source of variability [16], an artificial intelligence (AI)-assisted CT imaging system was used to quantify the severity of COVID-19 pneumonia. This system bases on a deep learning method for automatic segmentation and quantification of infection regions from chest CT scan [2]. The volume of infection (VOI) and the percentage of infection (POI) could be extracted from this system, and subsequently chest CT score could be calculated. Finally, in our study, lung injuries were quantified using three radiological parameters (VOI, POT and chest CT scores).

## Methods

### Study patients

We recruited 357 COVID-19 patients admitted to the Nanjing Public Health Medical Center (the Second Hospital of Nanjing) from July to August 2021. All the patients were linked to the transmission chain starting from Nanjing Lukou International Airport. The COVID-19 cases were confirmed by positive SARS-COV-2 nucleic acid tests. For the patients with SARS-COV-2 PCR cycle threshold (Ct) values less than 30, Delta variant infection was confirmed by sequencing. The patients were included if they: (1) fulfilled the definition of vaccination status in this study; (2) aged 18 years old or more; (3) have chest CT evaluation by artificial intelligence software. Since the study only focused on inactivated vaccines, patients that received other types of COVID-19 vaccines were excluded from analysis. The medical records, including demographic characteristics, vaccine status, laboratory parameters, comorbidities and clinical features, were collected from the electronic health record system. This study was approved by the ethics committee of the Second Hospital of Nanjing (2020-LS-ky003). Written informed consent was waived by the Ethics Commission.

### Definition of vaccination status and disease severity

Patients were divided into three groups according to their vaccination status: unvaccinated group; partially vaccinated group and fully vaccinated group. Unvaccinated groups were never received any COVID-19 vaccine before infectious with SARS-COV-2. While partially vaccinated group means patients who had only received one dose of vaccine at least 14 days before the diagnosed of COVID-19. Fully vaccinated patients were received two shots vaccines while the time interval between the second shot and illness onset was at least 14 days. To avoid ambiguity in definition, the patients who received their first dose within 14 days or their second dose within 14 days before the disease onset were not included in our study.

The severity of COVID-19 was defined according to the “Guideline of COVID-19 Diagnosis and Treatment (trial version 8)” issued by the National Health Council of China. Severe COVID-19 should meet one of the following criteria: (1) respiratory rate ≥ 30 breaths/min, (2) at rest, the oxygen saturation of fingers while breathing air ≤ 93%, (3) arterial partial pressure of oxygen (PaO2)/oxygen uptake concentration (FiO2) ≤ 300 mmHg. Critical COVID-19 cases were those who: (1) developed respiratory failure requiring mechanical ventilation; (2) had evidence of shock or other organ dysfunctions necessitating transferring to the intensive care unit (ICU) [3]. Other clinical conditions were grouped as non-severe COVID-19.

### Laboratory tests for SARS-COV-2

Nasopharyngeal swab specimens were immersed in cell preservation solution (X1003, Sansure Biotech, Hunan, China). The total nucleic acids were extracted from 200 μL cell preservation solution using an automated nucleic acid extraction system (BioPerfectus technologies company, Jiangsu, China). SARS-COV-2 nucleic acid was measured by quantitative reverse transcription polymerase chain reaction (qRT-PCR) kits (Sansure Biotech, Hunan, China). The cycle threshold (Ct) value from qRT-PCR was used to relatively represent the viral load. The levels of SARS-COV-2 IgM and IgG antibodies were measured using 2019-nCOV IgM and IgG antibody detection kits (BiOSCiENCE, Tianjin, China) targeting SARS-COV-2 spike receptor-binding domain (RBD). Serum samples were analyzed by an automated chemiluminescent immunoassay system (Axceed 260, BiOSCiENCE, Tianjin, China). A signal/cut-off (S/CO) value ≥ 1 was considered positive.

### Chest CT procedure

Chest computed tomography (CT) scans were generally performed every 3–5 days until resolution of clinical symptoms. Patients in the supine position were scanned with breath holding at the end of inhaling using a 64-CT scanner (Toshiba or Philips Healthcare). The scanning parameters were as follows: tube voltage 120 kV, tube current 110 mA, pitch 1.0, rotation time 0.5 s to 0.75 s, slice thickness 5 mm, and section thickness 1 mm or 1.5 mm for axial, coronal and sagittal reconstructions. During reading, the window width was 1200 HU and window level was − 600 HU.

### Assessment of lung lesions

The segmentation and quantitative analysis of COVID-19-associated pulmonary lesions in chest CT images were performed by uAI Discover-NCP (Shanghai United Imaging Intelligence Healthcare Co., Ltd.) [2]. A deep learning model named VB-Net was designed to segment both lung and lung infected regions from chin-section chest CT images. The segmentations include infection areas, the whole lung, lung lobes, and all the bronchopulmonary segments. A quantitative analysis procedure was used to automatically compute features from these extracted regions. As a result, quantitative metrics were generated to characterize infection regions, including the VOI and the POI in the whole lung, lung lobes, and the bronchopulmonary segments [2]. The Dice similarity coefficient was reported to be 91.6% ± 10.0% for AI segmentations and manual delineations, respectively, and the mean POI estimation error was 0.3% for the whole lung [2]. For each patient, the maximal value of POI in the whole lung was used as the COVID-19 pneumonia severity index (PSI). All chest CT scans with high PSI score were selected and evaluated by one radiologist and one experienced physician in pulmonary radiology independently. The evaluation results confirmed that the aforementioned chest CT scans selected by AI software contained severe lung lesions. In the case of disagreement, consensus was achieved by discussion.

In the present study, VOI and POI in the whole lung were used to represent the severity of the COVID-19 pneumonia. In additional, data of POI in the lung lobes were used to calculate the chest CT scores, a semi-quantitative method relatively representing the severity of pulmonary lesions. Following the principle described in previous studies [4, 5], each lung lobe was scored from 0 to 5 based on degree of involvement. No involvement was assigned a score of 0. Values of POI in the lobe < 5%, 5–25%, 26–49%, 50–75%, and > 75% were given scores of 1, 2, 3, 4, and 5, respectively. The score of each chest CT scan was the sum of 5 lobe scores, therefore could be ranged from 0 to 25. Together, this study used 3 parameters (VOI, POI in the whole lung, and the chest CT scores) to represent the severity of lung injury.

### Statistical analysis

Patients’ characteristics were assessed with standard descriptive statistics. Categorical values were presented by frequencies and percentages while medians and interquartile ranges (IQR) were used for continuous variables. As appropriate, comparisons were done using the Kruskal–Wallis test, Mann–Whitney U test, Pearson chi-square test, or Fisher’s exact test. Three continuous variables describing lung injury were selected as outcomes: VOI in the whole lung, POI in whole lung and the chest CT scores. Spearman’s rank correlation coefficient was used to measure correlation between two continuous variables. Univariate and multivariate generalized linear regression analysis between three outcomes and other variables which may influence them were included in this study, and the relationship was expressed with coefficients and 95% confidence intervals (95% CIs) due to the continuous outcomes. A subgroup analysis by stratifying age, sex, and comorbidity was performed. Two-tailed tests were performed, and a p value of < 0.05 was considered statistically significant. All analyses were performed using R software for Windows version 4.0.5 (https://www.r-project.org/).

## Results

### Characteristics of the patients

Of the 357 Delta COVID-19 patients included for analysis, 105 (29.4%), 72 (20.2%), and 180 (50.4%) were unvaccinated, partially vaccinated and fully vaccinated, respectively. The types of inactivated COVID-19 vaccines used were CoronaVac (Sinovac Biotech, Beijing, China), BBIBP-CorV (Sinopharm, Beijing, China), and KCONVAC (BioKangtai, Shenzhen, China), accounting for 73.3%, 26.5%, and 0.2% of the vaccination shots, respectively. The median age was 49 (IQR: 39–65) years old, and 108 (30.3%) of the patients were older than 60 years. There were no significant differences in sex, diabetes, asthma, autoimmune diseases, most clinical symptoms, and levels of C-reactive protein, lymphocytes, LDH, and D-dimer among patients with different vaccination status (Table 1) at the time of admission. Interleukin-6 level was significantly lower in fully vaccinated patients (P < 0.001, Table 1).

**Table1:** Characteristics of 357 Delta variant–infected patients with different vaccination statuses

	Unvaccinated (n = 105)	Partially Vaccinated (n = 72)	Fully Vaccinated (n = 180)	*P*-value
Demographics and clinical characteristics				
Sex				
Male	40 (38.1)	35 (48.6)	67 (37.2)	0.233
Female	65 (61.9)	37 (51.4)	113 (62.8)	
Age, years	67.00 [52.00, 74.00]	51.00 [39.75, 67.25]	44.00 [37.00, 52.00]	< 0.001
18–59	36 (34.3)	46 (63.9)	167 (92.8)	< 0.001
≥ 60	69 (65.7)	26 (36.1)	13 (7.2)	
With any comorbidity	45 (42.9)	23 (31.9)	33 (18.3)	< 0.001
Hypertension	34 (32.4)	18 (25.0)	23 (12.8)	< 0.001
Diabetes	13 (12.4)	7 (9.7)	9 (5.0)	0.069
Heart disease	7 (6.7)	2 (2.8)	2 (1.1)	0.029
Tumor	6 (5.7)	1 (1.4)	1 (0.6)	0.013
Asthma	4 (3.8)	0 (0.0)	2 (1.1)	0.143
Autoimmune diseases	1 (1.0)	1 (1.4)	2 (1.1)	1.000
Time from illness onset to hospitalization	3.00 [2.00, 5.00]	3.00 [1.00, 4.00]	2.00 [1.00, 4.00]	0.004
Symptoms				
Fever	38 (36.2)	26 (36.1)	48 (26.7)	0.148
Cough	50 (47.6)	35 (48.6)	94 (52.2)	0.743
Shortness of breath	7 (6.7)	3 (4.2)	2 (1.1)	0.029
Loss of smell or taste	2 (1.9)	2 (2.8)	11 (6.1)	0.212
Stuffy nose or runny nose	9 (8.6)	9 (12.5)	32 (17.8)	0.088
Pharyngeal discomfort	21 (20.0)	20 (27.8)	41 (22.8)	0.464
Blood laboratory findings				
C-reactive protein-mg/L	4.89 [0.50, 12.01]	6.77 [2.46, 20.12]	5.72 [2.20, 14.24]	0.177
> 10	31 (29.5)	32 (44.4)	63 (35.0)	0.126
Interleukin-6, pg/mL	16.93 [8.73, 28.58]	15.97 [4.77, 25.06]	5.92 [1.50, 13.57]	< 0.001
> 7	84 (80.8)	51 (71.8)	77 (43.0)	< 0.001
Lymphocyte count, × 10^9^/L	1.07 [0.90, 1.44]	1.29 [0.90, 1.69]	1.17 [0.88, 1.56]	0.210
< 0.8	21 (20.0)	14 (19.4)	28 (15.6)	0.560
LDH, IU/L	245.00 [214.00, 292.00]	244.50 [203.50, 282.75]	230.5 [197.75, 269.25]	0.068
> 245	52 (49.5)	36 (50.0)	72 (40.0)	0.186
D-dimer, mg/L	0.40 [0.27, 0.64]	0.41 [0.28, 0.62]	0.35 [0.21, 0.58]	0.083
> 0.55	35 (33.3)	21 (29.2)	48 (26.7)	0.481
Microbiological and serological data				
SARS-COV-2 IgM, S/CO	0.06 [0.03, 0.31]	0.37 [0.08, 1.26]	0.34 [0.11, 1.24]	< 0.001
IgM positive	16 (15.2)	20 (27.8)	55 (30.6)	0.012
SARS-COV-2 IgG (S/CO)	0.10 [0.05, 0.26]	0.52 [0.15, 2.24]	5.56 [2.35, 34.87]	< 0.001
IgG Positive	14 (13.3)	28 (38.9)	152 (84.4)	< 0.001
Viral load (Ct value)				
ORF1ab gene	23.00 [20.00, 27.00]	24.50 [20.00, 29.00]	22.00 [19.00, 27.00]	0.179
N gene	20.00 [17.00, 24.00]	20.50 [16.75, 26.00]	19.50 [15.00, 24.00]	0.166

Data were expressed as median (interquartile range, IQR) or n (%). As appropriate, a comparison between groups was made using Mann–Whitney U-test, Chi-Square test, or Fisher’s exact test. Ct, cycle threshold; SARS-COV-2, severe acute respiratory syndrome coronavirus 2. The Ct value was used to represent the viral load of SARS-COV-2 in the upper respiratory tract

**Fig. 1:**
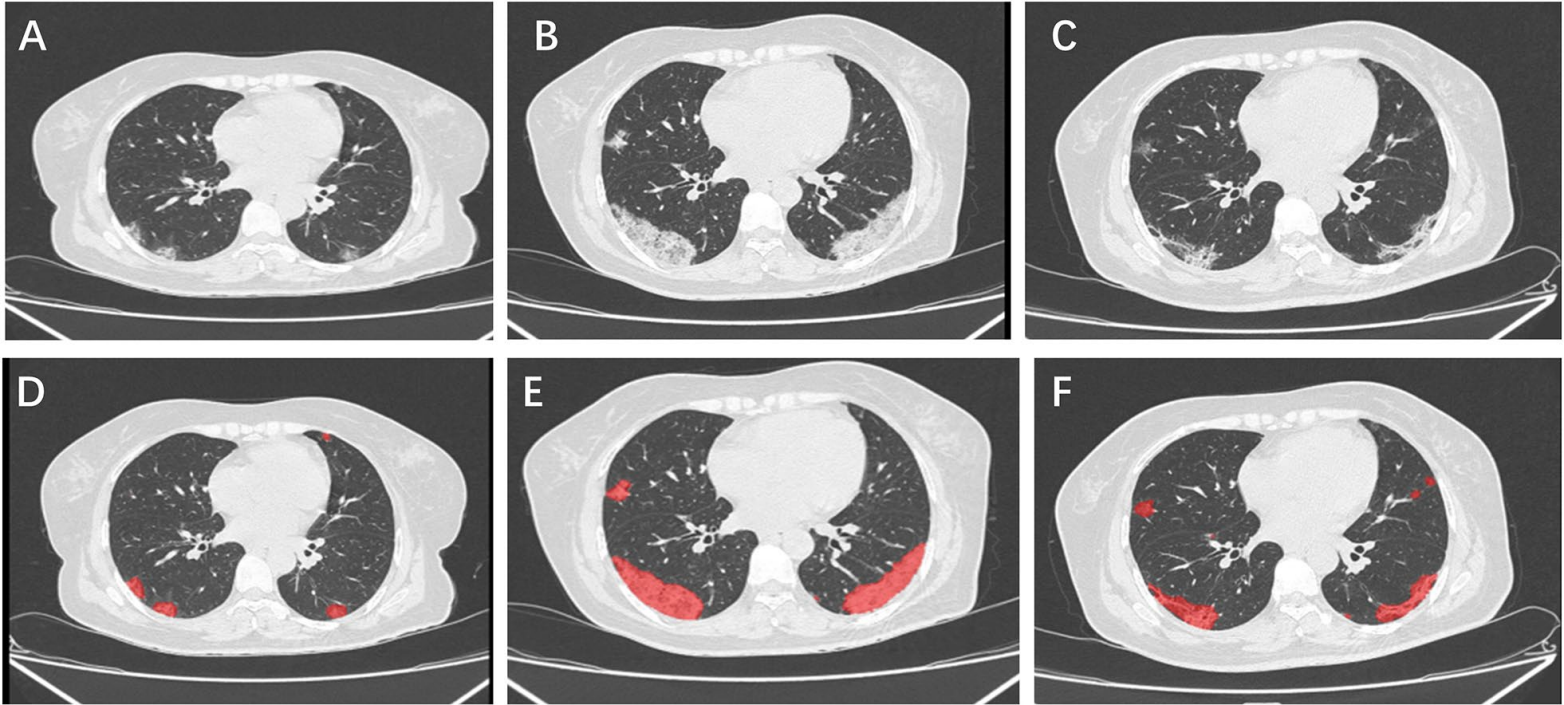
Illustration of Artificial Intelligence-Assisted CT imaging. Figures **A** to **C** were chest CT images of an unvaccinated patient during hospitalization. Chest CT of this patient was normal at admission, and on the third day of hospitalization showed traces of patchy ground-glass opacities on both lungs (**A**). A repeated chest CT on the 7th of hospitalization demonstrated substantial lesion progression. The most severe lung lesions were demonstrated by the chest CT scan obtained on the 11th day of hospitalization, with large patches of ground-glass opacities on both lungs (**B**). Follow-up chest CT scans performed on the 15th and 22nd day of hospitalization showed gradually improvement of the lung lesions. The last chest CT was completed on the 29th day of hospitalization, revealing a substantially resolution of lung lesions (**C**). Figures **D**–**F** demonstrated inflammatory areas on three CT scan images that were automatically demarcated by artificial intelligence (AI) software. The lung lesions demarcated by AI software were consistent with those delimited by visual inspection. The extent of lung injuries was presented as VOI and POI in the whole lung. For the three sequential chest CT scans of the patients, VOI (POI)in the whole lung was 47.1 cm^3^(1.3%); 523.6 cm^3^ (13.3%); 242.6 cm^3^ (6.2%), respectively.

There were significant differences in the VOI and POI in whole lung and the chest CT scores in patients with different vaccination status (P < 0.001, P < 0.001, P < 0.001 for three outcomes, respectively, Table 2). For all of the outcomes, the fully vaccinated group had less lung injury than unvaccinated patients (Table 2). Besides, the time from illness onset to most serious lung lesion was shorter in fully vaccinated group compared with unvaccinated group (6.00 days in fully vaccinated Vs 10.00 days in unvaccinated, P < 0.001, Table 2; an unvaccinated patient’s most severe lung lesions was on the 11th day of hospitalization, Fig. 1). There were also significant differences in the distribution of severe/critical and non-severe patients among the three groups (P < 0.001; Table 2).

**Table 2:** Severity of COVID-19 pneumonia assessed by artificial intelligence-assisted CT imaging

	Unvaccinated (n = 105)	Partially vaccinated (n = 72)	Fully vaccinated (n = 180)	*P*-value
VOI in the whole lung, cm^3^	222.40 [71.50, 455.90]	126.60 [27.75, 406.75]	39.90 [0.00, 150.02]	< 0.001
POI in the whole lung, %	7.60 [2.40, 14.60]	3.55 [1.12, 10.88]	1.20 [0.00, 5.10]	< 0.001
The chest CT scores	8.00 [5.00, 10.00]	6.00 [3.00, 9.00]	4.00 [0.00, 6.00]	< 0.001
Time from illness onset to most serious lung lesion	10.00 [7.00, 13.00]	8.00 [6.00, 10.00]	6.00 [3.00, 9.00]	< 0.001
severe/critical COVID-19	8 (7.6)	10 (13.9)	2 (1.1)	< 0.001

### Impact of inactivated COVID-19 vaccines on lung injuries

In univariable analysis (Table 3), compared with the unvaccinated group, the fully vaccinated group had a significantly decreased risk of lung injury {Coef[95%CI] = − 6.71 (− 8.64, − 4.78)}, P < 0.001; Coef(95%CI) = − 190.56 (− 247.4, − 133.72), P < 0.001, and Coef(95%CI) = − 3.58 (− 4.47,− 2.7), P < 0.001 for the POI and VOI in the whole lung, and the chest CT scores, respectively). A trend of less severe lung injuries was also seen in partially vaccinated patients (Coef (95%CI) = − 1.17 (− 3.58,1.24), P = 0.341; Coef (95%CI) = − 9.74 (− 80.56,61.08), P = 0.788; Coef(95%CI) = − 0.84 (− 1.94,0.26), P = 0.136 for three outcomes, respectively), however this was not statistically significant. Other factors that were associated with more lung injuries were older age, with underlying diseases, cough, higher levels of CRP, IL-6 and LDH, and lower levels of Lymphocyte count, SARS-COV-2 antibodies.

**Table 3:** Univariable analysis for factors associated with lung injury in delta variant–infected patients

	POI in the whole lung, %		VOI in the whole lung, cm^3^		The chest CT scores	
	Coefficient	P-value	Coefficient	P-value	Coefficient	P-value
Male (vs female)	− 0.87 (− 2.70,0.95)	0.348	43.30 (− 10.29,96.89)	0.114	− 0.48 (− 1.33,0.38)	0.275
Age ≥ 60 yeas (vs < 60 years)	8.00 (6.25,9.76)	< 0.001	228.00 (175.88,280.20)	< 0.001	4.18 (3.38,4.98)	< 0.001
Comorbidity (vs. No)	4.05 (2.11,5.98)	< 0.001	109.12 (51.80,166.44)	< 0.001	2.12 (1.22,3.02)	< 0.001
Vaccination status						
Unvaccinated	1		1		1	
Partially vaccinated	− 1.17 (− 3.58,1.24)	0.341	− 9.74 (− 80.56,61.08)	0.788	− 0.84 (− 1.94,0.26)	0.136
Fully vaccinated	− 6.71 (− 8.64, − 4.78)	< 0.001	− 190.60 (− 247.40, − 133.70)	< 0.001	− 3.58 (− 4.47, − 2.70)	< 0.001
Time from illness onset to hospitalization	0.20 (− 0.15,0.54)	0.264	5.71 (− 4.44,15.85)	0.271	0.06 (-0.10,0.22)	0.492
Fever	2.16 (0.25,4.07)	0.027	65.94 (9.63,122.24)	0.022	0.86 (− 0.03,1.76)	0.060
Cough	1.44 (− 0.34,3.23)	0.113	44.25 (− 8.19,96.68)	0.099	0.88 (0.05,1.71)	0.039
Shortness of breath	4.21 (− 0.72,9.15)	0.095	113.13 (− 32.43,258.68)	0.129	1.94 (− 0.37,4.25)	0.101
C-reactive protein	0.24 (0.18,0.29)	< 0.001	7.41 (5.82,8.99)	< 0.001	0.11 (0.08,0.13)	< 0.001
Interleukin-6	0.17 (0.14,0.20)	< 0.001	5.76 (4.90,6.63)	< 0.001	0.08 (0.06,0.09)	< 0.001
Lymphocyte count	− 4.17 (− 5.89, − 2.46)	< 0.001	− 127.09 (− 177.4, − 76.77)	< 0.001	− 2.29 (− 3.08, -1.50)	< 0.001
D-dimer	0.31 (− 0.58,1.19)	0.495	3.13 (− 22.94,29.19)	0.814	0.19 (− 0.23,0.60)	0.377
LDH	0.05 (0.04,0.06)	< 0.001	1.34 (1.02,1.66)	< 0.001	0.02 (0.02,0.03)	< 0.001
SARS-COV-2 IgM	− 1.84 (− 3.00, − 0.68)	0.002	− 55.56 (− 89.60, − 21.53)	0.002	− 1.03 (− 1.57, − 0.49)	< 0.001
SARS-COV-2 IgG	− 2.59 (− 3.34, − 1.84)	< 0.001	− 78.23 (− 100.23, − 56.23)	< 0.001	− 1.39 (− 1.73, − 1.05)	< 0.001
N gene	− 0.05 (− 0.19, 0.09)	0.521	− 2.03 (− 6.15, 2.10)	0.336	− 0.04 (− 0.10, 0.03)	0.250

In multivariable analysis, the effect of vaccination on pulmonary injuries was adjusted for age, sex, comorbidity, time from illness onset to hospitalization and SARS-COV-2 viral load base on clinical consideration. Since clinical parameters such as lymphocyte counts, CRP, IL-6 and LDH were more appropriate as an index of disease severity rather than risk factors, those variables not included in the multivariable regression analysis.

After adjusted for confounding factors, fully vaccination was still significantly associated with lower extent of lung injuries {Coef_adj_ [95%CI] = − 3.88(− 5.96, − 1.79}, P < 0.001; Coef_adj_(95%CI) = − 106.10(− 167.30, − 44.89), P = 0.001; and Coef_adj_(95%CI) = − 1.81(− 2.72, − 0.91), P < 0.001 for the three outcomes, respectively; Table 4). Of note, SARS-COV-2 viral load was not associated with the severity of the COVID-19 pneumonia in our patients (P = 0.334, P = 0.161, P = 0.188 for the three outcomes, respectively; Table 4).

**Table 4:** Multivariable analysis for factors associated with lung injury in delta variant–infected patients

	POI in the whole lung, %		VOI in the whole lung, cm3		The Chest CT scores	
	Adjusted coefficient	P-value	Adjusted coefficient	P-value	Adjusted coefficient	P-value
Male (vs female)	− 0.80(− 2.45,0.85)	0.342	46.51(− 1.84,94.86)	0.060	− 0.34(− 1.05,0.38)	0.356
Age ≥ 60 yeas (vs < 60 years)	0.16(0.10,0.22)	< 0.001	4.94(3.20,6.67)	< 0.001	0.11(0.08,0.13)	< 0.001
Comorbidity (vs. No)	0.62(− 1.36,2.59)	0.540	− 2.09(− 60.01,55.82)	0.944	0.03(− 0.82,0.89)	0.939
Vaccination status						
Unvaccinated	1		1		1	
Partially vaccinated	0.51(− 1.86,2.88)	0.671	34.07(− 35.40,103.53)	0.337	0.19(− 0.84,1.22)	0.722
Fully vaccinated	− 3.88(− 5.96, − 1.79)	< 0.001	− 106.10(− 167.30, 44.89)	0.001	− 1.81(− 2.72, − 0.91)	< 0.001
Time from illness onset to hospitalization	0.12(− 0.22,0.46)	0.480	4.52(− 5.41,14.46)	0.373	0.02(− 0.12,0.17)	0.745
Viral load	− 0.07(− 0.20,0.07)	0.334	− 2.87(− 6.88,1.13)	0.161	− 0.04(− 0.10,0.02)	0.188

### Subgroup analysis

Subgroup analyses were performed for the three outcomes stratified by sex, age, and comorbidity status (Additional file 1: Tables S1, S2, S3). Likewise, partially vaccination was not associated with any reduction of pulmonary injuries in the subgroup analyses. In fully vaccinated patients, variation of protection could be found within subgroups. Generally, the extent of reduction of pulmonary injuries was more profound in fully vaccinated patients with older age, having underlying diseases, and being female sex, as demonstrated by relatively larger absolute values of adjusted coefficients (Additional file 1: Tables S1, S2, S3). Nevertheless, for patients with underlying diseases, the reduction of pulmonary injury in fully vaccinated patients was statistically significant only when pulmonary lesions were evaluated by chest CT scores {Coef_adj_[95%CI] = − 2.70(− 4.83, − 0.57), P = 0.015}. This suggested that chest CT scores may be more sensitive than POI and VOI in the whole lung, when evaluating different treatment strategies for COVID-19 pneumonia. Finally, when patients with severe/critical COVID-19 diseases were excluded from analysis, fully vaccination was still significantly associated with less pulmonary injuries {Coefadj[95%CI] = − 3.24(− 4.93, − 1.54)}, P < 0.001; Coefadj(95%CI) = − 97.44(− 147.33, − 47.55), P = 0.001; and Coefadj(95%CI) = − 1.65(− 2.51, − 0.79), P < 0.001 for the POI and VOI in the whole lung, and the chest CT scores, respectively; Additional file 1: Table S4).

## Discussion

As a typical respiratory virus, SARS-COV-2 infection leads to viral pneumonia. Respiratory failure is the leading cause of death in COVID-19 patients [6]. Chest CT scan is not only a sensitive tool for diagnosing COVID-19 pneumonia but also a useful method for evaluating the severity and extent of disease [7–9]. Our present study found that fully vaccinated patients had the least lung impairments evaluated by any of the three radiological parameters. In addition, shorter duration from illness onset to peak lung injury was observed in fully vaccinated patients, suggesting that convalescent stage may come earlier in fully vaccinated patients. When adjusted for age, sex, comorbidity, time from illness onset to hospitalization and SARS-COV-2 viral load that may potentially influence the severity of the COVID-19 pneumonia, fully vaccination was still significantly associated with lower extent of lung injuries. However, protection against lung injury was not found in partially vaccinated patients. The results in our study are consistent with the findings in previous studies that fully vaccination with inactivated COVID-19 but not partially vaccination protect against severe COVID-19 [10, 11, 17–19].

In many infectious diseases, high burden of pathogens is generally associated with more advanced diseases and poorer prognosis [20–23]. However, in COVID-19 cases, this positive association is controversial [16, 24–27], probably because SARS-COV-2 may not be the only contributor to the disease progression. Host immune response may be an important regulator of the clinical course of COVID-19. Aberrant inflammation could lead to severe deterioration of COVID-19 pneumonia, whereas proper immune responses lead to immune clearance of infection while maintaining minimal lung injury [28, 29]. In our study, SARS-COV-2 viral load was neither associate with vaccination status nor associated with the severity of the COVID-19 pneumonia. The findings at least suggest that higher viral load at the early stage of Delta variant infection does not always predict more severe COVID-19.

Previous study suggested that vaccine efficacy may be influenced by the sex, age and comorbidities [30, 31]. Our study showed that fully vaccination results in more profound reduction of lung injury in elderly patients, patients with underlying diseases, and female patients. The finding implies that those groups of patients may benefit more from COVID-19 vaccine. Nevertheless, fully vaccination is required for those patients because there was no reduction of pulmonary injuries in partially vaccinated patients in the subgroup analysis. Of note, statistical differences were most likely to be observed when chest CT score was used to represent the severity of the COVID-19 pneumonia. More studies are needed to explore what is the best radiological parameter representing severity of lung injury. Finally, when only non-severe patients were included in the analysis, our study was still able to demonstrated that fully vaccination was associated with protection of lung injury.

Although the degree of COVID-19 pneumonia on chest CT scan could be a promising outcome parameter for evaluating the effect of COVID-19 vaccine, it would not be expected to replace the current method in vaccine studies. Frequent chest CT scans are required for accurately capturing the most severe COVID-19 pneumonia. In real world clinical practice, this is very difficult to be achieved, especially in patients with mild and stable diseases. In additional, radiological manifestations of COVID-19 pneumonia is not always correlated with the severity of the disease. Exacerbation of preexisting medical conditions by COVID-19 could also threaten the patient’ s life.

In conclusion, our study demonstrated that fully vaccination but not partially vaccination could significantly protect lung injury. Elderly patients, patients with underlying diseases, and female patients may benefit more from COVID-19 vaccines. Our study provides additional evidence to encourage a full course of vaccination.

## Supplementary Information

**Additional file 1: Table S1.** Subgroup analysis of impact of inactivated COVID-19 vaccines on VOI in the whole lung. **Table S2.** Subgroup analysis of impact of inactivated COVID-19 vaccines on POI in the whole lung. **Table S3.** Subgroup analysis of impact of inactivated COVID-19 vaccines on the chest CT scores. Multivariable analysis for factors associated with lung injury in non-severe Delta variant–infected patients. **Table S4.** Multivariable analysis for factors associated with lung injury in non-severe Delta variant–infected patients.

## Data Availability

The datasets used and/or analysed during the current study are available from the corresponding author on reasonable request.
