# Supplementary material for: Impact of inactivated COVID-19 vaccines on lung injury in B.1.617.2 (Delta) variant-infected patients
Source: Ann Clin Microbiol Antimicrob. 2023 Mar 21;22:22. doi: 10.1186/s12941-023-00569-z (PMC10029781; doi:10.1186/s12941-023-00569-z)
Supplement: Supplementary file 1 — Additional file 1: Table S1. Subgroup analysis of impact of inactivated COVID-19 vaccines on VOI in the whole lung. Table S2. Subgroup analysis of impact of inactivated COVID-19 vaccines on POI in the whole lung. Table S3. Subgroup analysis of impact of inactivated COVID-19 vaccines on the chest CT scores. Multivariable analysis for factors associated with lung injury in non-severe Delta variant–infected patients. Table S4. Multivariable analysis for factors associated with lung injury in non-severe Delta variant–infected patients. [file 12941_2023_569_MOESM1_ESM.docx]

***Appendix***

**Additional file Tables**

**Additional file 1: Table S1. Subgroup analysis of impact of inactivated COVID-19 vaccines on VOI in the whole lung**

|  | Unvaccinated (Ref.) | Partially vaccinated | | | | | Fully vaccinated | | | | |
| --- | --- | --- | --- | --- | --- | --- | --- | --- | --- | --- | --- |
|  | N | N | Coef | P | Coef_adj_ | P_adj_ | N | Coef | P | Coef_adj_ | P_adj_ |
| All patients | 105 | 72 | -9.74 (-80.56,61.08) | 0.788 | 34.07(-35.4,103.53) | 0.337 | 180 | -190.56 (-247.4, -133.72) | <0.001 | -106.10(-167.30, -44.89) | 0.001 |
| Age-18-59 | 36 | 46 | -12.72(-93.89,68.45) | 0.759 | -13.27(-93.04, 66.49) | 0.745 | 167 | -76.60(-143.62, -9.57) | 0.026 | -62.90(-129.32, 3.51) | 0.065 |
| Age≥60 | 69 | 26 | 136.88(6.36, 267.41) | 0.042 | 126.54(-4.47, 257.55) | 0.061 | 13 | -258.06(-429.56, -86.56) | 0.004 | -266.93(-440.31, -93.54) | 0.003 |
| Without comorbidity | 69 | 49 | -50.41(-130.17, 29.35) | 0.217 | -5.90(-80.57,68.78) | 0.877 | 147 | -196.91(-260.37, -133.45) | <0.001 | -119.96(-182.69, -57.24) | <0.001 |
| With comorbidity | 45 | 23 | 81.83(-61.46,225.13) | 0.266 | 85.47(-71.29,242.23) | 0.288 | 33 | -142.91(-271.04, -14.79) | 0.031 | -145.43(-308.22,17.35) | 0.083 |
| Female | 65 | 37 | 0.75(-80.20, 81.71) | 0.985 | 38.19 (-40.84,117.21) | 0.345 | 113 | -210.00(-271.19, -148.80) | <0.001 | -123.89 (-192.67, -55.11) | <0.001 |
| Male | 40 | 35 | -23.69(-151.46,104.08) | 0.717 | 26.04(-100.46, 152.53) | 0.687 | 67 | -157.47(-267.77, -47.16) | 0.006 | -95.62(-210.30, 19.06) | 0.104 |

**Additional file 1: Table S2. Subgroup analysis of impact of inactivated COVID-19 vaccines on POI in the whole lung**

|  | Unvaccinated (Ref.) | Partially vaccinated | | | | | Fully vaccinated | | | | |
| --- | --- | --- | --- | --- | --- | --- | --- | --- | --- | --- | --- |
|  | N | N | Coef | P | Coef_adj_ | P_adj_ | N | Coef | P | Coef_adj_ | P_adj_ |
| All patients | 105 | 72 | -1.17(-3.58,1.24) | 0.341 | 0.51(-1.86,2.88) | 0.671 | 180 | -6.71 (-8.64, -4.78) | <0.001 | -3.88(-5.96, -1.79) | <0.001 |
| Age-18-59 | 36 | 46 | -0.77(-3.41, 1.88) | 0.570 | -0.81(-3.42,1.81) | 0.547 | 167 | -2.37 (-4.56, -0.19) | 0.034 | -2.12(-4.30, 0.06) | 0.058 |
| Age≥60 | 69 | 26 | 3.49(-1.09, 8.07) | 0.138 | 3.30(-1.33,7.94) | 0.166 | 13 | -9.34 (-15.36, -3.32) | 0.003 | -9.08(-15.22, -2.95) | 0.005 |
| Without comorbidity | 69 | 49 | -1.86(-4.42, 0.70) | 0.156 | -0.33(-2.71,2.05) | 0.786 | 147 | -6.44(-8.47, -4.40) | <0.001 | -4.09(-6.09, -2.09) | <0.001 |
| With comorbidity | 45 | 23 | 0.86 (-4.38, 6.10) | 0.748 | 1.43(-4.32,7.18) | 0.628 | 33 | -5.71( -10.40, -1.03) | 0.019 | -5.46(-11.43,0.52) | 0.077 |
| Female | 65 | 37 | -0.30 (-3.54, 2.94) | 0.857 | 1.36 (-1.79, 4.51) | 0.399 | 113 | -8.21 (-10.66, -5.76) | <0.001 | -4.48 (-7.22, -1.74) | 0.002 |
| Male | 40 | 35 | -1.47(-5.04, 2.10) | 0.420 | -0.49(-4.09, 3.10) | 0.788 | 67 | -4.26 (-7.34, -1.18) | 0.008 | -2.97(-6.24, 0.29) | 0.076 |

**Additional file 1: Table S3. Subgroup analysis of impact of inactivated COVID-19 vaccines on the chest CT scores**

|  | Unvaccinated (Ref.) | Partially vaccinated | | | | | Fully vaccinated | | | | |
| --- | --- | --- | --- | --- | --- | --- | --- | --- | --- | --- | --- |
|  | N | N | Coef | P | Coef_adj_ | P_adj_ | N | Coef | P | Coef_adj_ | P_adj_ |
| All patients | 105 | 72 | -0.84 (-1.94,0.26) | 0.136 | 0.19(-0.84,1.22) | 0.722 | 180 | -3.58 (-4.47, -2.7) | <0.001 | -1.81(-2.72, -0.91) | <0.001 |
| Age-18-59 | 36 | 46 | 0.06 (-1.48, 1.61) | 0.938 | 0.01 (-1.54,1.55) | 0.994 | 167 | -1.09 (-2.37, 0.19) | 0.095 | -1.07(-2.36, 0.21) | 0.104 |
| Age≥60 | 69 | 26 | 0.71 (-0.70, 2.11) | 0.326 | 0.63 (-0.78, 2.03) | 0.383 | 13 | -4.52 (-6.37, -2.67) | <0.001 | -4.42 (-6.27, -2.56) | <0.001 |
| Without comorbidity | 69 | 49 | -0.91(-2.27, 0.45) | 0.191 | 0.03(-1.17,1.24) | 0.956 | 147 | -3.33(-4.41, -2.25) | <0.001 | -1.80(-2.81, -0.79) | 0.001 |
| With comorbidity | 45 | 23 | -0.31(-2.19, 1.58) | 0.751 | 0.24(-1.81,2.28) | 0.822 | 33 | -3.18 (-4.87, -1.49) | <0.001 | -2.70(-4.83, -0.57) | 0.015 |
| Female | 65 | 37 | -0.49 (-1.90, 0.91) | 0.491 | 0.41(-0.88, 1.70) | 0.535 | 113 | -4.22(-5.28, -3.16) | <0.001 | -2.17(-3.29, -1.05) | <0.001 |
| Male | 40 | 35 | -0.91 (-2.68, 0.86) | 0.313 | -0.05(-1.75, 1.66) | 0.958 | 67 | -2.54 (-4.07, -1.01) | 0.001 | -1.33 (-2.88, 0.21) | 0.093 |

**Additional file 1: Table S4. Multivariable analysis for factors associated with lung injury in non-severe Delta variant–infected patients**

|  | **POI in the whole lung, %** | | **VOI in the whole lung, cm3** | | **The Chest CT scores** | |
| --- | --- | --- | --- | --- | --- | --- |
|  | Mul-Coef | Mul -p | Mul -Coef | Mul -p | Mul -Coef | Mul -p |
| Male (vs female) | -0.41(-1.76,0.94) | 0.551 | 45.36(5.73,84.99) | 0.026 | -0.29(-0.98,0.39) | 0.401 |
| Age≥60 yeas (vs <60 years) | 0.14(0.09,0.19) | <0.001 | 4.40(2.98,5.83) | <0.001 | 0.10(0.08,0.13) | <0.001 |
| Comorbidity (vs. No) | -0.66(-2.31,0.99) | 0.434 | -30.80(-79.39,17.79) | 0.215 | -0.35(-1.19,0.49) | 0.410 |
| Vaccination Status |  |  |  |  |  |  |
| Unvaccinated | 1 |  | 1 |  | 1 |  |
| Partially vaccinated | -0.44(-2.45,1.58) | 0.671 | -18.87(-78.23,40.48) | 0.534 | -0.15(-1.17,0.88) | 0.778 |
| Fully vaccinated | -3.24(-4.93, -1.54) | <0.001 | -97.44(-147.33, -47.55) | 0.001 | -1.65(-2.51, -0.79) | <0.001 |
| Time from illness onset to hospitalization | -0.04(-0.32,0.23) | 0.754 | -1.32(-9.47,6.82) | 0.751 | -0.03(-0.17,0.12) | 0.727 |
| Viral load | -0.02(-0.13,0.10) | 0.788 | -0.63(-3.93,2.66) | 0.706 | -0.02(-0.08,0.03) | 0.434 |
